# The complete chloroplast genome of *Grateloupia turuturu Yamada*

**DOI:** 10.1080/23802359.2020.1723449

**Published:** 2020-02-06

**Authors:** Hongbin Han, Yan Li

**Affiliations:** a First Institute of Oceanography, Ministry of Natural Resources, Qingdao, China;; b Laboratory of Marine Ecology and Environmental Science, Pilot National Laboratory for Marine Science and Technology, Qingdao, China;; c Laboratory of Marine Ecology and Environmental Science, Qingdao National Laboratory for Marine Science and Technology, Qingdao, China

**Keywords:** Chloroplast genome, *Grateloupia turuturu Yamada*, phylogenetic analysis, *Grateloupia filicina*, *Grateloupia taiwanensis*

## Abstract

In this study, we sequenced and annotated the complete chloroplast genome of *Grateloupia turuturu Yamada* (GenBank accession number: MN853877). The total length of the chloroplast genome is 188,547 bps, including 196 protein-encoding genes, 23 tRNA genes and 3 rRNA. The complete chloroplast genome of *G. turuturu* is 30.68% C + G, which is lower than that of A + T. The phylogenetic tree, which is based on core genes, shows that *G. turuturu* is clustered into the *Grateloupia* clade and has close genetic relationships with algae *Grateloupia filicina* and *Grateloupia taiwanensis*. These data will provide more information to understand the phylogenetic status of *G. turuturu*.

*G. turuturu* belongs to the phylum Rhodophyta, class Florideophyceae, order Halymeniales, family *Halymeniaceae*, and the genus *Grateloupia*. It mainly grows on rocks or in tide pools in the low tidal zone and is widely distributed along the coast of China, especially in the Yellow and Bohai Seas (Xia 2004). *G. turuturu* is rich in proteins, carbohydrates, vitamins and other minerals making it a subsidiary food (Fujiwara-Arasaki et al. 1984; Rui et al. 2006). *G. turuturu* has antiviral and anti-tumor activities, which makes it potentially interesting for pharmaceutical and medical developments (Simon-Colin et al. 2002; Hellio et al. 2004). In addition, *G. turuturu* could also be the raw material for agar or carrageenan (Shanmugam and Mody 2000; Fu et al. 2011).

*G. turuturu* was collected from the Qinhunangdao coastal area (39°49′54.69″N, 119°31′30.50″E) and cultured in the laboratory with VSE medium at 20 °C under a light intensity of 100 μmol^−2^ s^−1^ (Zhou et al. 2016a, 2016b). The specimens were preserved at the Marine Ecology Research Center of the First Institute Oceanography, Ministry of Natural Resources in Qingdao (Accession number: DXWGZ06). Approximately 5 g of fresh algae was harvested for cpDNA isolation using an improved extraction method (Chen et al. 2011). After DNA isolation, 1 μg of purified DNA was fragmented to construct short-insert libraries (insert size of 430 bps) according to the manufacturer’s instructions (Illumina), and then sequenced on the Illumina Hiseq 4000 (Borgstrom et al. 2011) at Shanghai BIOZERON Co., Ltd. Prior to assembly, the Illumina raw reads were filtered. The chloroplast genome of *G. turuturu* was reconstructed using a combination of the Pacbio Sequel data and the Illumina Hiseq data via SPAdes v3.10.1 (Antipov et al. 2016).

The shape of the chloroplast genome of *G. turuturu* is a double-stranded closed loop and has the GenBank accession number of MN853877. The complete chloroplast genome sequence of *G. turuturu* is 188,547 bps long with a C＋G composition of 30.68%. The genome contains 196 protein-coding genes as well as a number of non-coding genes, including 26 tRNA genes and 3 rRNA genes (*rrn23*, *rrn16* and *rrn5*). All of the coding genes begin with ATG except for *chlI*, *dnaB*, *infC*, *psbY*, *rbcS*, *rps8* and *ycf63*, which begin with GTG. The termination codons for *accA*, *acsF*, *apcE*, *atpE*, *bas1*, *carA*, *ccs1*, *dnaK*, *dsbD*, *groEL*, *grx*, *ompR*, *pbsA*, *petA*, *petF*, *petM*, *psaF*, *psaL*, *psaM*, *psbB*, *tsf*, *ycf16*, *ycf20*, *ycf60* and *ycf80* are TAG. On the other hand, *accB*, *cfxQ*, *chlI*, *dnaB*, *infB*, *psbL*, *rne*, *rpoC2*, *rps18*, *rps19*, *secA*, *trpA* and *ycf63* have termination codons of TGA. The rest 158 genes end with TAA.

To determine the phylogenetic position of *G. turuturu*, 24 other complete chloroplast genome sequences were obtained from the Genebank database. A phylogenetic tree was constructed based on core genes. Maximum likelihood (ML) methods were performed for the phylogenetic analysis using PhyML 3.0, and the bootstrap was 1000. The phylogenetic tree (Figure 1) shows that *G. turuturu* clustered into the *Grateloupia* clade and has close genetic relationships with algae *Grateloupia filicina* and *Grateloupia taiwanensis.*

**Figure 1.**
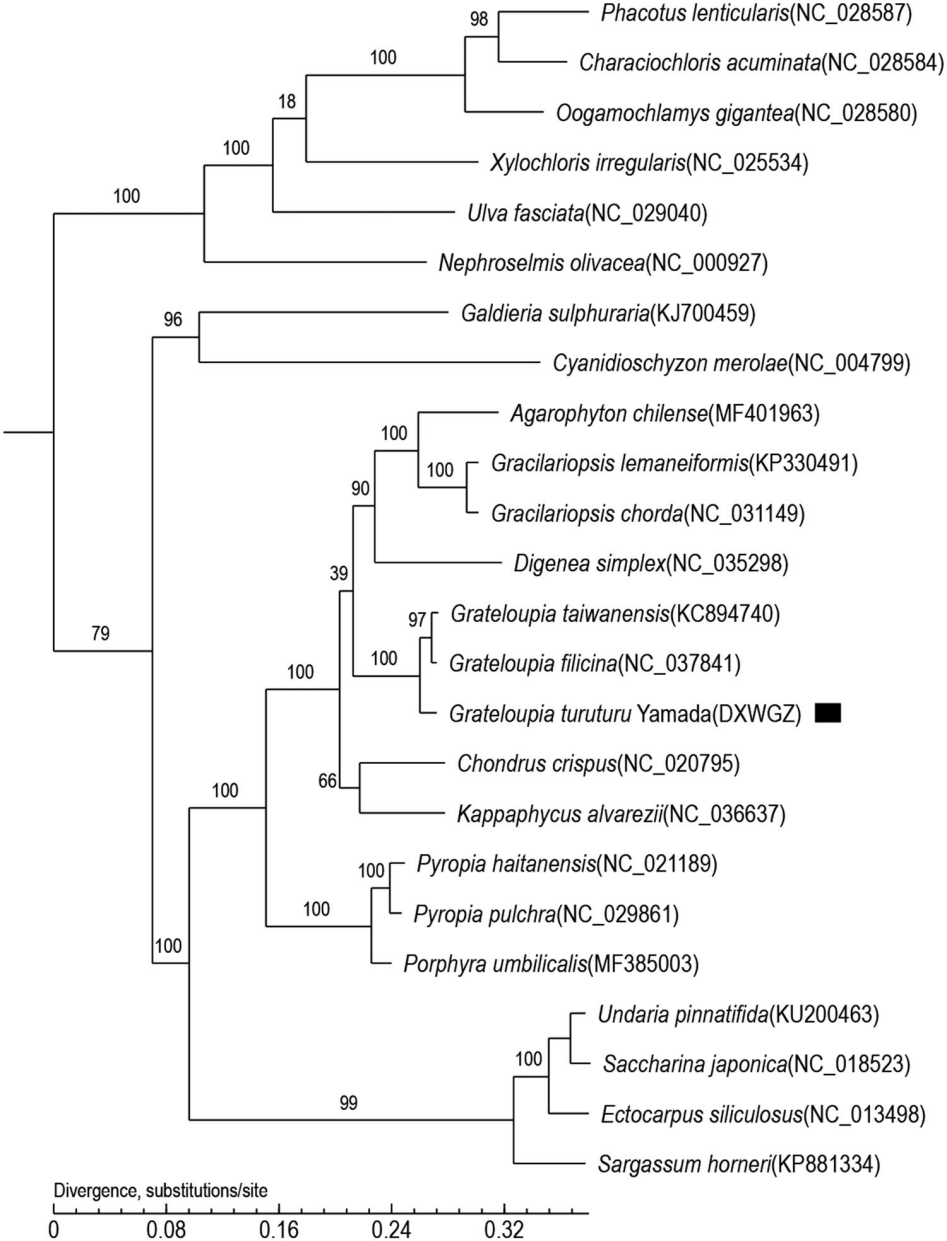
Maximum-likelihood (ML) tree based on the complete chloroplast genome sequences of 25 species. The numbers on the branches are bootstrap valuese.
